# High Quantum Yield Green-Emitting Carbon Dots for Fe(ІІІ) Detection, Biocompatible Fluorescent Ink and Cellular Imaging

**DOI:** 10.1038/s41598-017-15054-9

**Published:** 2017-11-01

**Authors:** Waheed Ullah Khan, Deyin Wang, Wei Zhang, Zuobin Tang, Xinlong Ma, Xin Ding, Shanshan Du, Yuhua Wang

**Affiliations:** 1 School of Physical Science and Technology, Lanzhou University, Lanzhou, 730000 P. R. China; 2 School of Basic Medical Sciences, Lanzhou University, Lanzhou, 730000 P. R. China

## Abstract

In the present work, we reported the luminescence of a green-emitting carbon dots (CDs) synthesized via solid state reaction method using diammonium hydrogen citrate and urea as a starting materials. The obtained green-emitting CDs shows strong absorption in the 350–450 nm region and gives intense green emission (λ_max_ = 537 nm) with quantum yield as high as 46.4% under 420 nm excitation. The obtained green-emitting CDs also demonstrates high photo-stability, which is evidenced by the fact that its emission intensity almost has no change under irradiation by a 365 nm UV lamp for 2 hours. Moreover, the obtained green-emitting CDs shows high sensitivity and selectivity for the detection of Fe^3+^, and their emission intensity response towards Fe^3+^ ions is highly linear (R^2^ = 0.995) over the concentration range from 25 to 300 µM, which could provide an effective platform for detection of Fe^3+^. Mostly important, we further demonstrate that such photoluminescent green-emitting CDs exhibits low toxicity and are biocompatible for use with in cellular imaging. Combining with low cytotoxicity, good water solubility and excellent luminescence properties, green-emitting CDs could be used as a biocompatible fluorescent ink in future applications.

## Introduction

As a new comer of fluorescent nanoparticle, carbon dots (CDs) continue to fascinate a mankind because of their outstanding physiochemical properties such as high luminescence, low-toxicity, surface modification flexibility, super resistance to photobleaching, good biocompatibility and excellent water solubility^1–4^. CDs is intensely recognized for their potential applications in bio-imaging, optoelectronics, photocatalysis, light-emitting diodes, sensing and energy conversion/storage devices^5–11^. These excellent physiochemical properties and their numerous applications make CDs a promising alternative to organic dyes, polymer dots and semiconductors quantum dots that have been extensively studied over the past decades^12,13^. However, the toxicity, poor water solubility and photobleaching, complex synthesis procedure and low quantum yield (QY) of the latter materials (organic dyes, polymer dots and semiconductors quantum dots) have limited their practical applications^14–17^.

After a careful reviewing of the existing literatures, it is found that many different types of materials and approaches have been used to synthesize CDs. The reported raw materials vary from laboratory-produced chemicals to natural products, and approaches include laser ablation, pyrolysis, electrochemical oxidation, hydro/solvo-thermal reactions, and microwave treatment *et al*.^15,18–22^. However, most of the resultant carbon dots emit blue and blue-green, and few are found to emit in longer wavelengths^10,23–26^. In addition, the methods mentioned above are not able to produce large quantity of product. Moreover, carbon dots suffer from the difficulty in producing high quantum yield (QY) and long wavelength (>500 nm) emissive products, which are considered as a major disadvantage of CDs. Therefore, it is urgent to develop a new way to synthesize carbon dots that can emit a longer emission beyond blue region, so as to enrich the luminescence properties and diversify its applications.

Herein, we report for the first time highly fluorescent green-emitting carbon dots through a facile solid state reaction method with quantum yield as high as 46.4% under 420 nm excitation. The resulting green-emitting CDs can emit bright and stable green luminescence at high salt concentration and show good photostability under continuous 365 nm UV lamp irradiation for 2 hours. The obtained green-emitting CDs possess excitation-dependent emission and short monoexponential fluorescent lifetime. Besides, the green-emitting CDs is shown to be able to detect Fe^3+^ ions with good sensitivity and selectivity over the concentration range from 25 to 300 µM. Because of their outstanding photoluminescence properties and low-cytotoxicity, green-emitting CDs is particularly suitable for biocompatible fluorescent ink and also fluorescent probe for cellular imaging. For their easy preparation and unique optical properties make these CDs promising for numerous applications in modern science and technology.

## Results and Discussion

### Photoluminescent properties of the green-emitting CDs

Different combinations of materials with diammonium hydrogen citrate were used to synthesize carbon dots, however, only the carbon dots synthesized by using diammonium hydrogen citrate with urea were found give green emission, and all the rest give blue emission (see supporting information Figure S1). As a result, urea with diammonium hydrogen citrate are ultimately selected due to their products yielded green emission with high quantum yield, indicating that green emissive products with highly quantum yield are dominate to synthesize carbon dots.

**Figure 1:**
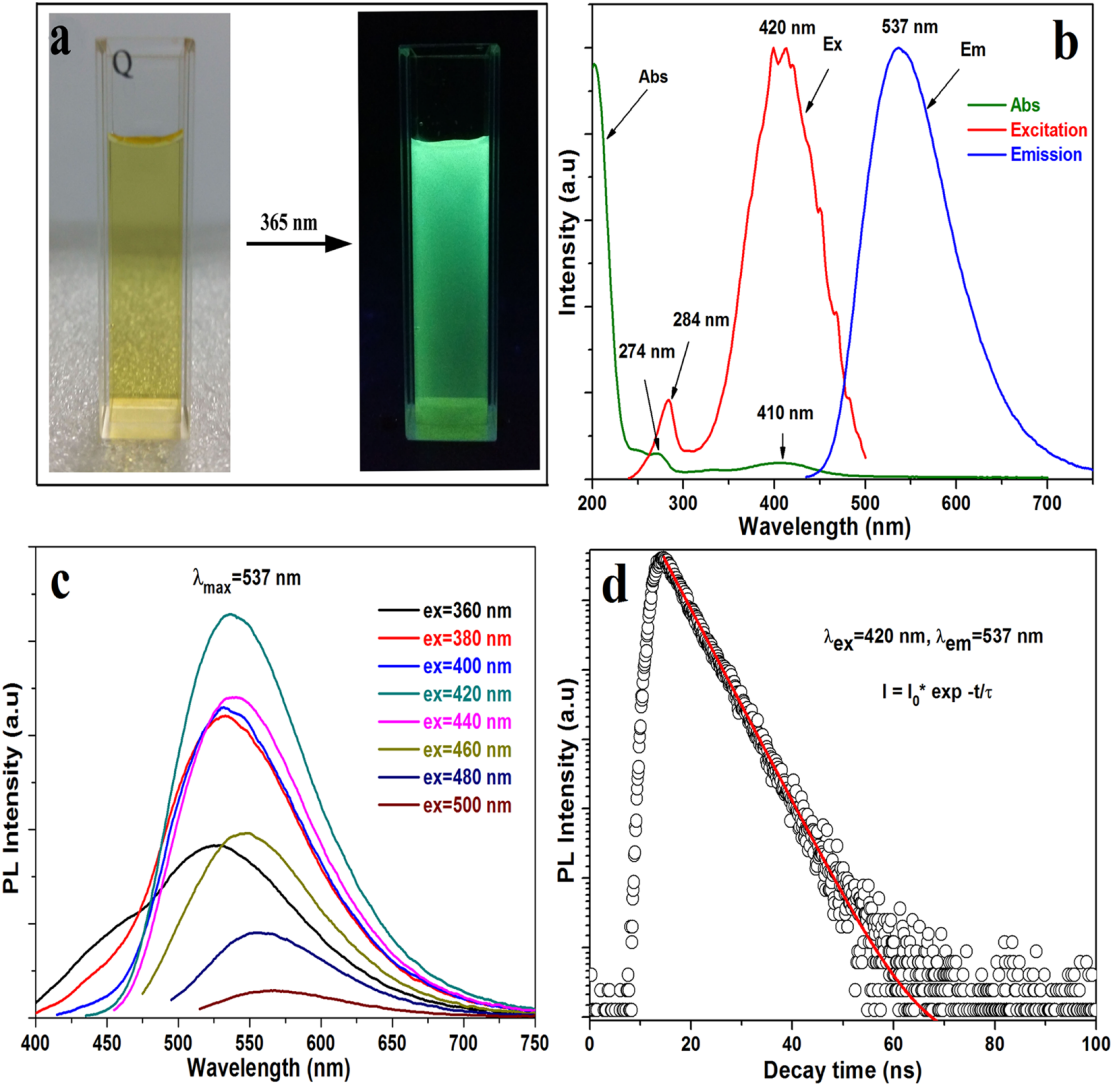
(**a**) Photos of the green-emitting CDs under sunlight (left) and UV irradiation (right). (**b**) UV-Vis absorption, excitation and emission spectra of the obtained green-emitting CDs. (**c**) The emission spectra of green-emitting CDs under different excitations. (**d**) Decay curve of the prepared green-emitting CDs under 420 nm exictation.

**Figure 2:**
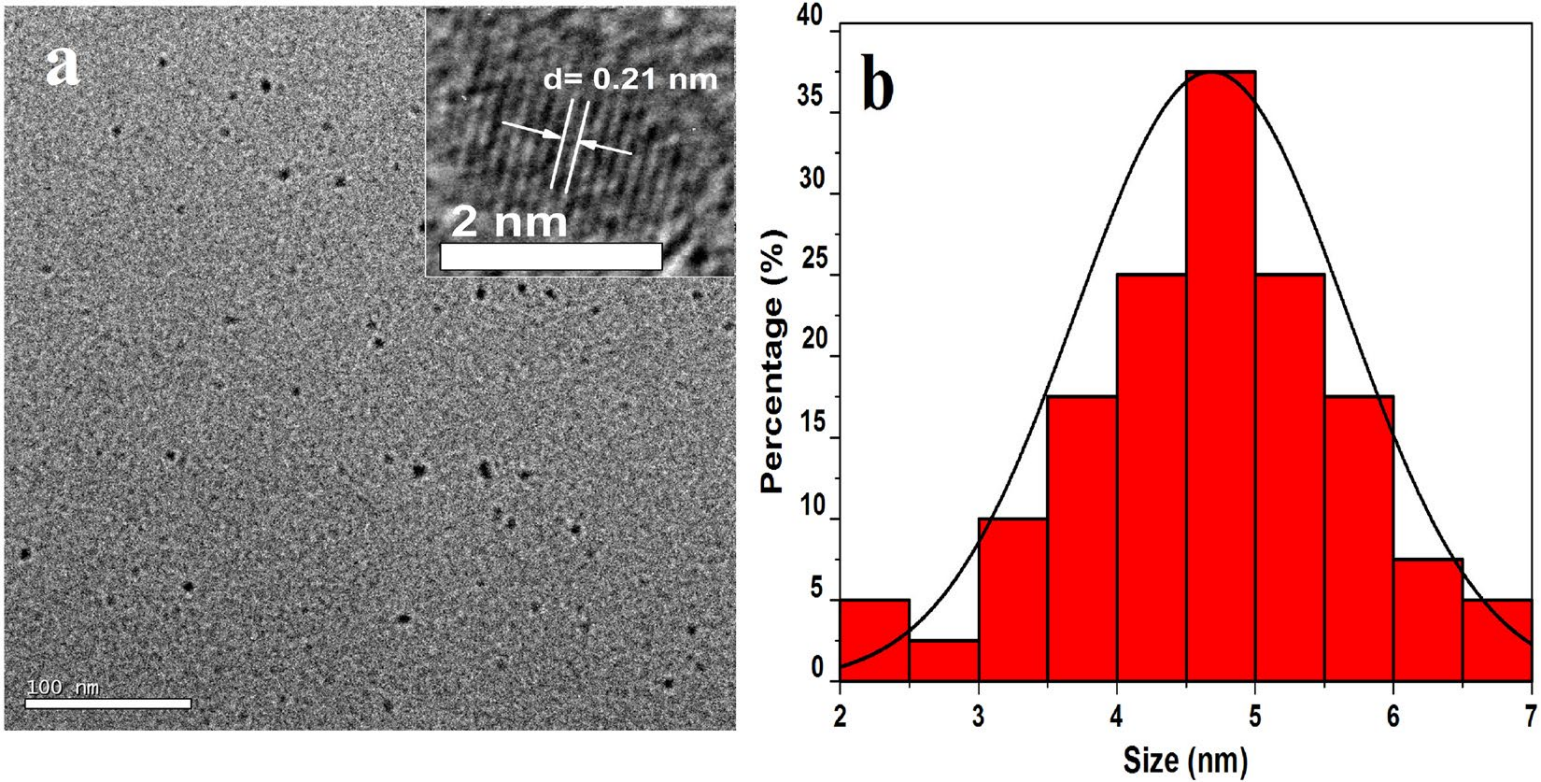
(**a**) The TEM and (inset) HRTEM image (**b**) and size distribution of the green-emitting CDs.

The obtained carbon dots are yellow under day light irradiation but green under a 365 nm UV lamp irradiation, which can be seen in Fig. 1a. The absorption and photoluminescence (PL) properties of the aqueous green-emitting CDs is characterized (Fig. 1b) by the ultraviolet-visible (UV-Vis) and PL spectrometer. In the UV-Vis spectrum, it can be seen clearly that there are two absorption peaks appeared at 274 and 410 nm. The absorption peak at 274 nm is attributed to carbonic core center and the peak catered at 410 nm is ascribed to surface or molecule center^27^. Under 420 nm excitation, the carbon dots yield green emission at 537 nm (see Fig. 1b). The excitation spectrum obtained by monitoring the green emission at 537 nm is consistent with the result presented in the absorption spectrum. This behavior also indicates that there are at least two types of excitation energy trapping on the surface of CDs^19^. In additon, it is found that the green-emitting CDs exhibit excitation-dependent emission as shown in Fig. 1c. When the excitation wavelenght is increased from 360 to 500 nm with the increment of 20 nm, the emission peak is red shifted from 525 to 570 nm. The excitation-dependent emission behavior of greem-emitting CDs is related to be the non-uniform particle size and distribution of different surface states^7,28,29^. TEM image confirms that the size of the particle is not very uniform (see Fig. 2). The QYs of the green-emitting CDs is determined to be respectable in either organic solvent (e.g. ethanol, 46.4%, Figure S2) or DI water (15.5%, Figure S3) under 420 nm excitation by using a rhodamine 6 G (95%) solution as a reference. To best of our knowledge, this is the highest QY value for the green-emitting CDs reported so far. Figure 1d presents the decay curve of the obtained green-emitting CDs with excitation and emission wavelengths in 420 and 537 nm respectively. The decay curve can be fitted by a single exponential function:

1 $${\rm{N}}({\rm{t}})={{\rm{A}}}_{0}\,exp(-{\rm{t}}/{\rm{\tau }})$$

The decay lifetime was determined to be 7.2 ns, and such a short lifetime indicates that the luminescence mechanism of our obtained green-emitting CDs is the radiative recombination nature^19^.

High stability is one of the important factors in nano-size flourescent materials, which plays a significant role to demonstrate their application in various fields. The photo-stability and salt (KCl) effect on the green-emitting CDs have been investigated. It is confirmed that green-emitting CDs is highly stable under continuous irradiation of a 365 nm UV lamp for two hours (supporting information Fig. S4a). Furthermore, the prepared green-emitting CDs has favorable ionic strength at high salt (KCl) concentration of about 2 M (supporting information Fig. S4b). Moreover, the green-emitting CDs is confirmed to be relatively stable over the pH range from 2 to 9.5 as shown in the (supporting information Fig. S5).

The obtained green-emitting CDs in aqueous state exhibits a long-term homogeneous phase without any noticeable precipitation at room temperature. The TEM images and size distribution of the prepared green-emitting CDs are displayed in Fig. 2a, from which it can be seen clearly that the obtained particles are scattered in regular manner and possess closely spherical pattern. The inset HRTEM image demonstrate the crystallinity of the green-emitting CDs with a lattice parameter of 0.21 nm, which agrees with (100) lattice fringes of graphite^30^. The size of the obtained green-emitting CDs is not very uniform and it is distributed in the ranges from 2 to 7 nm. The average size of the green-emitting CDs is 4.6 nm as displayed in Fig. 2b.

**Figure 3:**
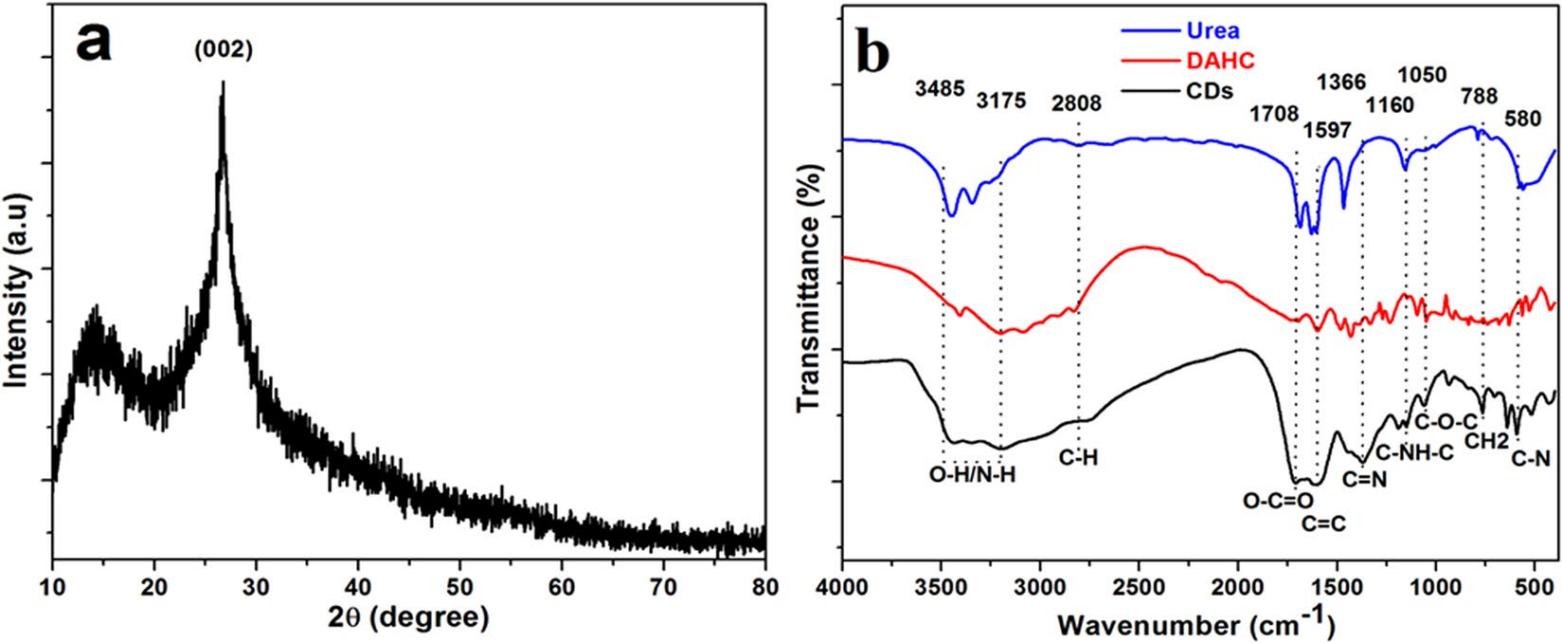
(**a**) The XRD profile of the prepared green-emitting CDs. (**b**) FT-IR spectrum of Urea, diammonium hydrogen citrate (DAHC) and obtained green-emitting CDs.

The XRD profile of the green-emitting CDs shows (002) peak centred at 26° as shown in the Fig. 3a. The result is similar to that of the graphite, showing highly disordered carbon atoms^31,32^. The structure and composition of green-emitting CDs were characterized by FT-IR spectroscopy. As shown in Fig. 3b, the synthesized green-emitting CDs show main absorption bands related to OH/NH stretching vibration at 3435 to 3175 cm^−1^. These functional groups improve the hydrophilicity and stability of green-emitting CDs in aqueous systems^3,33^. The weak peak at 2808 cm^−1^ is related to the C-H band stretching vibration. The peaks at 1708 and 1597 cm^−1^ are ascribed to the O-C = O and C = C stretching vibrations, respectlively^23^. Moreover, the CH/CN band is starching at 1366 cm^−1^ on the surface of the green-emitting CDs. The characteristic stretch band of the amine H-N bond is at 1150 cm^−1^. The IR bands appear within the range of 1050 to 580 cm^−1^, which correspond to the stretching vibration of C-O, CH_2_ and C-N, respectively^34^. The unsaturated carbon bonds were further examined by the Raman spectrum, which shows the feature of D band at 1375 cm^−1^ and G band at 1596 cm^−1^ (supporting information, Fig. S6). The ratio of the relative intensity of D band and G band (*I*_*D*_ */I*_*G*_) is about 0.96, indicating the disorder nature of the green-emitting CDs with poor crystalline framework^32^.

**Figure 4:**
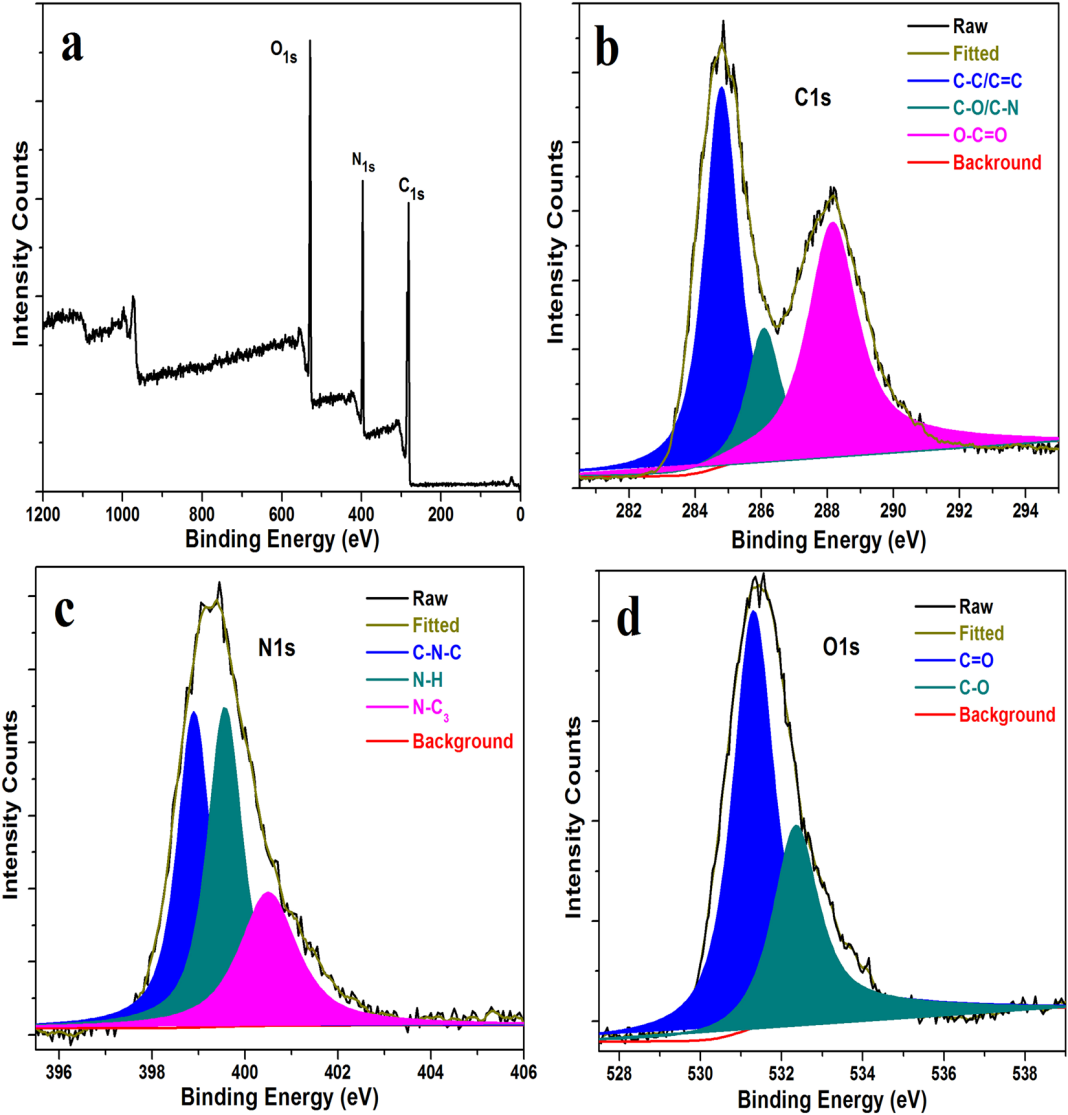
(**a**) Full XPS spectra of the obtained CDs. (**b**–**d**) High-resolution XPS spectra of the C 1 s, N 1 s and O1s of the obtained green-emitting CDs, respectively.

X-ray photoelectron spectroscopy (XPS) measurements were performed for the surface elemental analysis of green-emitting CDs. The wide XPS spectrum of the green-CDs as shown in Fig. 4a clearly reveals that carbon (284.6 eV), nitrogen (398.8 eV), and oxygen (531.3 eV) are presented at the surface of green-emitting CDs. In the expanded XPS spectra (Fig. 4b), the C1s band can be deconvoluted into three peaks, which were assigned to sp2 carbons (C = C, 284.5 eV), sp3 (C-O/C-N, 286.1 eV), carboxyl carbons (O-C = O, 288.1 eV). The N1s band can be deconvoluted into three peaks (Fig. 4c), which were corresponded to (C-N-C, 398.8 eV), amino N (N-H, 399.5 eV) and (C_3_-N, 400.5 eV). The oxygen containing groups are also presented in the surface of the green-emitting CDs (Fig. 4d), the O1s band contains two peaks associated to sp^2^ (C = O, 531.3 eV) and sp^3^ (C-O, 532.3 eV)^35,36^.

## Application of green-emitting CDs

### Detection of Fe^3+^ in an aqueous solution

**Figure 5:**
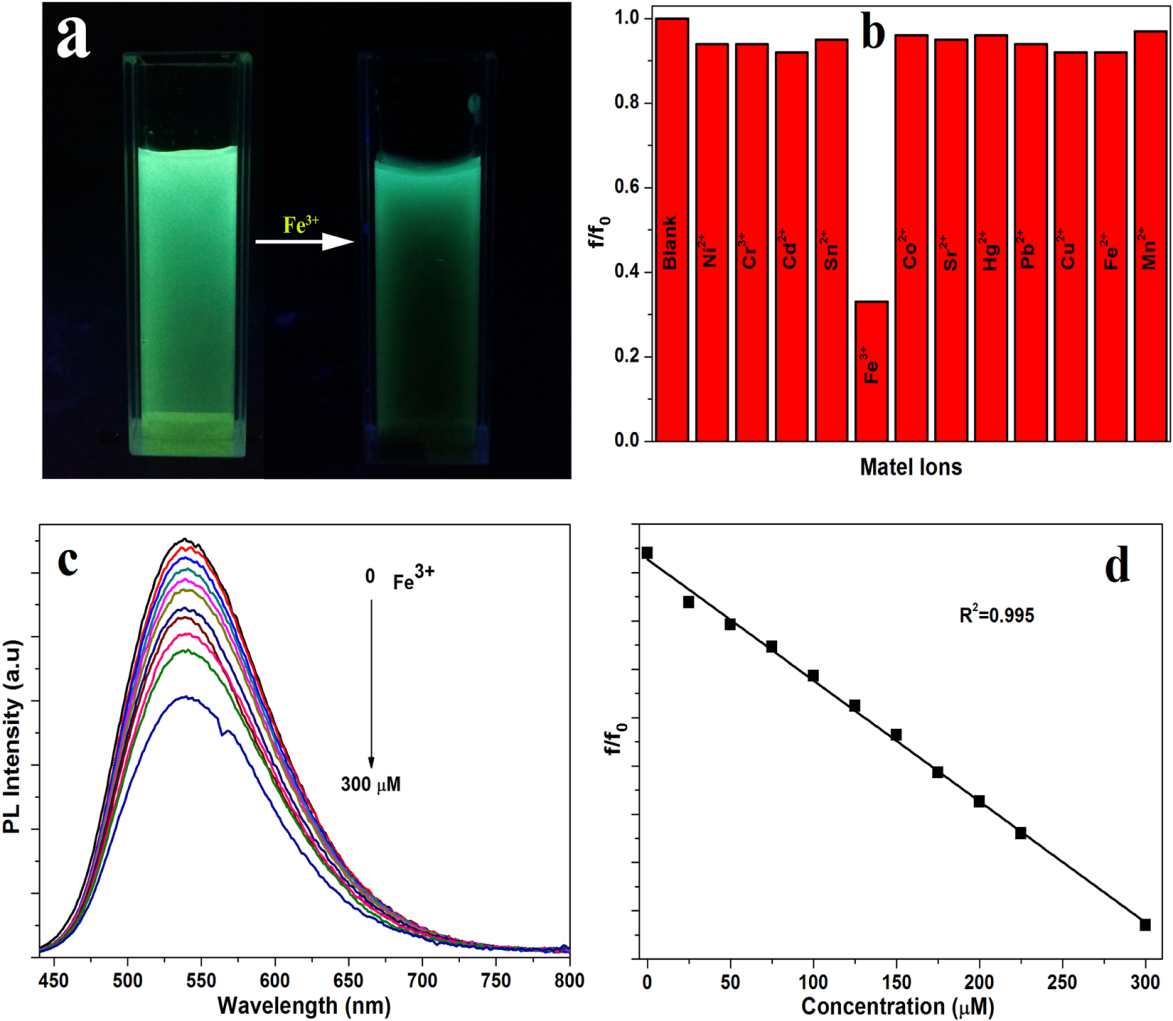
(**a**) photographs of the green-emitting CDs without and with Fe ^3+^ ions under 365 nm UV lamp irradiation. (**b**) Emission intensity of the green-emitting CDs after adding different metal ions with same concentration. (**c**) The photoluminescence spectra of green-emitting CDs with different concentration of Fe^3+^ from 25 to 300 µM. (**d**) The dependence of f/f_0_ on the concentration of Fe^3+^.

The Fe^3+^ plays an important role in life due to their essential function in oxygen metabolism, oxygen transport, transfer of electrons and catalytic procedures^37^. Recently, carbon fluorescent dots have been used as a fluorescent probe for heavy metal ions, showing good sensitivity and selectivity towards different metal ions. The photographs in Fig. 5a show the emission of green-emitting CDs before and after adding Fe^3+^ ions. Many common metal ions have been used for detection (Fig. 5b), but only Fe^3+^ is able to quench the fluorescence of green-emitting CDs, suggesting the green-emitting CDs have a potential to detect Fe^3+^ ions via luminescence measurements. The observed fluorescent quenching by Fe^3+^ ions could be attributed to its strong interaction with surface groups of green-emitting CDs, which facilitate the transformation of photoelectron from CDs to Fe^3+^ ions^7,38^. Figure 5c shows the fluorescent spectra of the green-emitting CDs aqueous solution containing different concentration of Fe^3+^ ions: 25, 50, 75, 100, 125, 150, 175, 200, 225 and 300 µM. The photoluminescence spectra are decreasing by regular manner adding the different concentration of Fe^3+^ (excitation wavelength 420 nm). The ratios between (f/f_0_) and Fe^3+^ concentration have a good linear relationship with correlation coefficient R^2^ of 0.995 as shown in Fig. 5d. The limit of detection (LOD) is calculated to be 19 µM using the 3 times σ/K ratio. The observed results clearly illustrating that the green-emissive CDs have a good sensitivity and selectivity towards Fe^3+^ ions, which can be used as a practical application. The analytical performances of recent reported sensors for the detection of Fe^3+^ are summarized in (Table 1).

**Table 1:** Analytical performances of recent reported sensors for the detection of Fe^3+^.

Synthetic method	Precursor	Linear range	Emission range/QYs %	Limit of detection (LOD)	Reference
Hydrothermal method	alginic acid and ethanediamine	0.0–0.05 mM	Blue/0.7	10.98 μM	39
	cellulose fibers	25–250 μM	Blue/32.2	0.96 μM	40
	L-glutamate	0–50 μM	Blue/17.8	4.67 μM	41
	potatoes	1.0–5.0 μM and 5.0–50.0 μM	Blue/15	0.025 μM	42
	α-lipoic acid, sodium hydroxide and ethylenediamine	0–500 μM	Blue/54.4	4 μM	7
	dopamine and ethanediamine	50–300 μM	Blue-green/9.4	10.8 µM	43
Electrochemical synthesis	graphite rods	0–1 µM	Blue/NA	2 nm	44
	ethanol	1–80 μM and 80–800 μM	Blue/10.04	0.04 µM	45
	Graphite electrode	10–200 µM	Blue/11.2	1.8 µM	46
Hummers method	GO nanosheets	14.32–143.2 μM	Blue/0.7	17.9 μM	47
Microwave irradiation	Chitosan, glacial acetic acid and EDA	0.179–32.22	Blue/20.1	0.179 µM	48
Solid-state reaction	Diammonium hydrogen citrate and urea	0–300 µM	Green/46.4	19 µM	This work

### Biocompatible fluorescent ink

**Figure 6:**
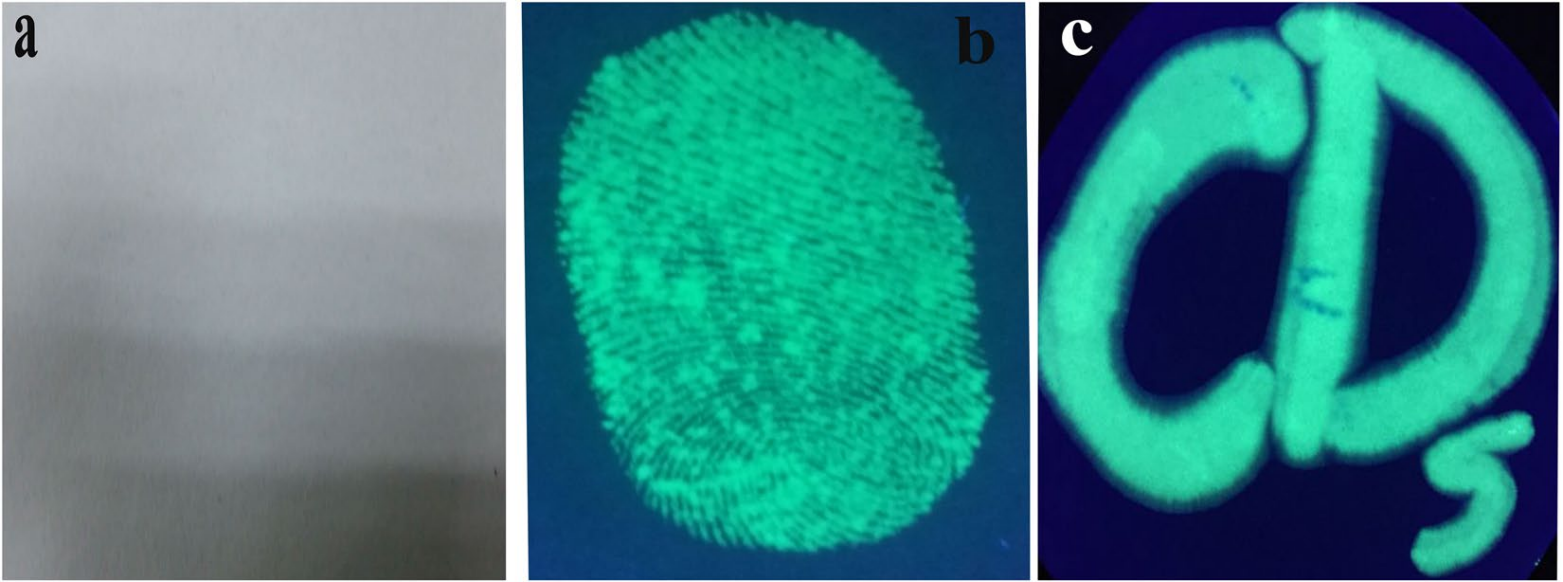
(**a**) Green-emitting CDs formed fingerprint under daylight (**b**) and 365 nm UV lamp irradiation. (**c**) Fluorescent characters on filter paper under UV excitation.

The low/non-toxicity at high concentration of green-emitting CDs indicates that it can possibly be used as a new type of biocompatible fluorescent ink. Figure 6a and b, show the green-emitting CDs based fluorescent fingerprint on filter paper under sun light and UV lamp (365 nm) irradiation. Figure 6c shows the fluorescent characters on filter paper irradiated by 365 nm UV beam. The carbon dots based fluorescent ink doesn’t contaminate fingers and can be easily washed off from water^4,27^.

### Cellular imaging in HeLa cells

**Figure 7:**
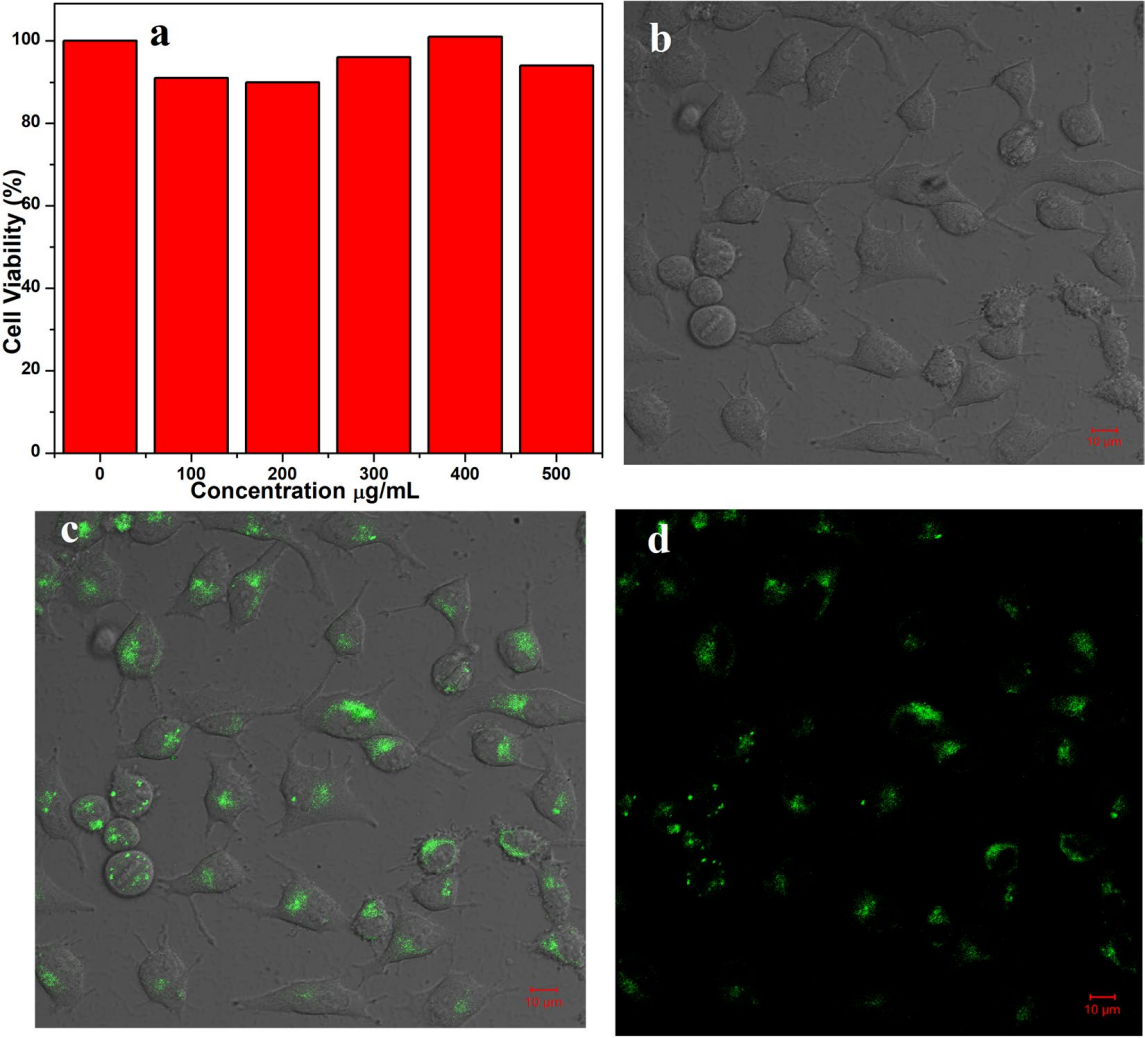
(**a**) Cellular toxicity assessment of green-emitting CDs. (**b**) Bright field image (**c** and **d**) Confocal fluorescent images of the green-emitting CDs under 405 nm laser excitation.

Carbon dots have a promising candidate for biological applications because of their non/low toxicity, good stability and highly luminescent materials^49–51^. The cytotoxicity of the green-emitting CDs was evaluated by using a standard MTT assay with HeLa cells^8^. As shown in the Fig. 7a, the average cell viability is above 90% with concentrations ranging from 100 to 500 µg/mL for 24 hours. The result suggests that the green-emitting CDs has very low cytotoxicity and good biocompatibility with high concentration about 500 µg/mL. Figure 7b shows the bright field image of the HeLa cells. After incubating the green-emitting CDs to HeLa cells for imaging, cells emitted a remarkable green fluorescence (Fig. 7c and d) and showed good morphology under confocal laser scanning microscopy. The fluorescence signals reveal that green-emitting CDs is mainly inside the cell membrane, suggesting that the green-emitting CDs can pass through cell membrane and enter into the cells cytoplasm. These results indicate good cell permeability of the green-emitting CDs towards HeLa cells, which further confirm the merits of the green-emitting CDs.

## Conclusion

In this work, we report the optical properties and possible applications of a green-emitting CDs synthesized by solid state reaction approach. Under 420 nm excitation, the obtained CDs emits bright green emission with quantum yield of 46.4%, which is the highest QY value for the green-emitting CDs reported so far. Besides, the obtained green-emitting CDs is proved to have favorable ionic strength, high stability, excellent water solubility and excitation dependent emission. The synthesized carbon dots have been demonstrated the potential to be used as a sensor of Fe^3+^ ions, fluorescent based-ink and cell marker. The low cost, biocompatibility, and low-toxicity of the green-emitting CDs and their distinct PL properties indicate that green-emitting CDs could potentially be synthesized on an industrial scale and could be used for numerous possible applications.

## Methods

### Materials

Diammonium hydrogen citrate, urea, L-cystiene, HD-met-oH, glycine, L-glutathione (GSH) and the salts (PbCl_2_, Cr(NO_3_)_3_, CuCl_2_, HgCl_2_, NiCl_2_, FeCl_2_, FeCl_3_, CdCl_2_, CoCl_2_, SnCl_2_, HgCl_2_, MnCl_2_ and SrCl_2_) were purchased from Sinopharm Chemical Reagent Co., Ltd, Rhodamine 6 G (99%) was purchased from Adamas Reagent Co., Ltd. All the chemicals were of analytical grade and used without further purification. The deionzed water was used throughout the experiments.

### Synthesis of green-emitting CDs

For a typical synthesis process diammonium hydrogen citrate (0.2 g) and urea (0.2 g) were grinded homogeneously in agate morter, and then the obtained mixture was heated in an oven at 180 °C for 1 hour in air. After the reaction was over, the resulting product was dissolved in water. The obtained solution was purified in a centrifuge at high speed (12000 rpm, 5 min) to remove the large particles. Afterword, the solution was further filtered via 0.22 µm filter membrane to obtain the pure CDs. The obtained solution of CDs was dispersed in deionize water for further characterizations.

### Characterizations

The morphology and size of the samples was observed by (FEI Tecnai F30 transmission electron microscope (TEM) operating at 300 kV). Photoluminescence (PL) and photoluminescence excitation (PLE) spectra were recorded using a Fluorolog-3 Spectrofluorometer equipped with a 450 W Xenon light source. Ultraviolet-visible (UV-Vis) absorption spectra were recorded using a Lambda 950, Perkin-Elmer (USA) UV-Vis-NIR spectrophotometer. The phase purity of fluorescent CDs was analyzed by X-ray diffraction (XRD) using a Bruker D2 PHASER X-ray Diffractometer with graphite monochromator using Cu Kα radiation (λ = 1.54056 Å), operating at 30 kV and 15 mA. Fourier transform infrared spectroscopy (FT-IR) was recorded a Nicolet 670 spectrophotometer at room temperature. X-ray photoelectron spectra (XPS) were acquired using a (XPS, PHI- 5702, Physical Electronics) with a monochromatic Al Kα irradiation source. The PL decay curves were measured by a FLS-920T fluorescence spectrophotometer with an nF900 nanosecond flash lamp as the light source. Raman spectroscopy was measured using a (JY-HR800 micro-Raman spectroscopy) with a 532 nm wavelength of YAG laser.

## Electronic supplementary material

Supporting information

## References

[1] Zheng Y (2015). A facile approach for the synthesis of highly luminescent carbon dots using vitamin-based small organic molecules with benzene ring structure as precursors. RSC Advances.

[2] Lan M (2015). A carbon dot-based fluorescence turn-on sensor for hydrogen peroxide with a photo-induced electron transfer mechanism. Chemical Communications.

[3] Gao Z-h, Lin Z-z, Chen X-m, Zhong H-p, Huang Z-y (2016). A fluorescent probe based on N-doped carbon dots for highly sensitive detection of Hg2 + in aqueous solutions. Analytical Methods.

[4] Zhu S (2013). Highly photoluminescent carbon dots for multicolor patterning, sensors, and bioimaging. Angewandte Chemie.

[5] Zhang Y-Q (2012). One-pot synthesis of N-doped carbon dots with tunable luminescence properties. Journal of Materials Chemistry.

[6] Algarra M (2014). Carbon dots obtained using hydrothermal treatment of formaldehyde. Cell imaging in vitro. Nanoscale.

[7] Ding H, Wei J-S, Xiong H-M (2014). Nitrogen and sulfur co-doped carbon dots with strong blue luminescence. Nanoscale.

[8] Ding H, Yu S-B, Wei J-S, Xiong H-M (2016). Full-Color Light-Emitting Carbon Dots with a Surface-State-Controlled Luminescence Mechanism. ACS Nano.

[9] Fernandes D, Krysmann MJ, Kelarakis A (2015). Carbon dot based nanopowders and their application for fingerprint recovery. Chemical Communications.

[10] Jiang K (2015). Red, Green, and Blue Luminescence by Carbon Dots: Full-Color Emission Tuning and Multicolor Cellular Imaging. Angewandte Chemie International Edition.

[11] Zhang Y-Q, Ma D-K, Zhang Y-G, Chen W, Huang S-M (2013). N-doped carbon quantum dots for TiO2-based photocatalysts and dye-sensitized solar cells. Nano Energy.

[12] Wu C, Chiu DT (2013). Highly Fluorescent Semiconducting Polymer Dots for Biology and Medicine. Angewandte Chemie International Edition.

[13] Zhou D (2013). Conducting the Temperature-Dependent Conformational Change of Macrocyclic Compounds to the Lattice Dilation of Quantum Dots for Achieving an Ultrasensitive Nanothermometer. ACS Nano.

[14] Dong Y (2013). Carbon-based dots co-doped with nitrogen and sulfur for high quantum yield and excitation-independent emission. Angewandte Chemie.

[15] De B, Karak N (2013). A green and facile approach for the synthesis of water soluble fluorescent carbon dots from banana juice. RSC Advances.

[16] Jaiswal A, Ghosh SS, Chattopadhyay A (2012). One step synthesis of C-dots by microwave mediated caramelization of poly(ethylene glycol). Chemical communications.

[17] Ding C, Zhu A, Tian Y (2014). Functional Surface Engineering of C-Dots for Fluorescent Biosensing and *in Vivo* Bioimaging. Accounts of Chemical Research.

[18] Qin X, Lu W, Asiri AM, Al-Youbi AO, Sun X (2013). Microwave-assisted rapid green synthesis of photoluminescent carbon nanodots from flour and their applications for sensitive and selective detection of mercury(II) ions. Sensors and Actuators B: Chemical.

[19] Yang Z (2014). Nitrogen-doped, carbon-rich, highly photoluminescent carbon dots from ammonium citrate. Nanoscale.

[20] Sun D (2013). Hair fiber as a precursor for synthesizing of sulfur- and nitrogen-co-doped carbon dots with tunable luminescence properties. Carbon.

[21] Shi L (2016). Carbon dots with high fluorescence quantum yield: the fluorescence originates from organic fluorophores. Nanoscale.

[22] Ramanan V (2016). Outright Green Synthesis of Fluorescent Carbon Dots from Eutrophic Algal Blooms for *In Vitro* Imaging. ACS Sustainable Chemistry & Engineering.

[23] Jiang K (2015). Bright-Yellow-Emissive N-Doped Carbon Dots: Preparation, Cellular Imaging, and Bifunctional Sensing. ACS Applied Materials & Interfaces.

[24] Kim E, Koh M, Lim BJ, Park SB (2011). Emission Wavelength Prediction of a Full-Color-Tunable Fluorescent Core Skeleton, 9-Aryl-1,2-dihydropyrrolo[3,4-b]indolizin-3-one. Journal of the American Chemical Society.

[25] Liu S, Zhao N, Cheng Z, Liu H (2015). Amino-functionalized green fluorescent carbon dots as surface energy transfer biosensors for hyaluronidase. Nanoscale.

[26] Wang H (2017). Excitation wavelength independent visible color emission of carbon dots. Nanoscale.

[27] Xu M (2014). A green heterogeneous synthesis of N-doped carbon dots and their photoluminescence applications in solid and aqueous states. Nanoscale.

[28] Wang C (2015). A hydrothermal route to water-stable luminescent carbon dots as nanosensors for pH and temperature. Carbon.

[29] Li W (2013). Simple and Green Synthesis of Nitrogen-Doped Photoluminescent Carbonaceous Nanospheres for Bioimaging. Angewandte Chemie International Edition.

[30] Chen BB (2016). A large-scale synthesis of photoluminescent carbon quantum dots: a self-exothermic reaction driving the formation of the nanocrystalline core at room temperature. Green Chemistry.

[31] Qu S, Wang X, Lu Q, Liu X, Wang L (2012). A Biocompatible Fluorescent Ink Based on Water-Soluble Luminescent Carbon Nanodots. Angewandte Chemie International Edition.

[32] Dong Y (2014). Graphene quantum dots, graphene oxide, carbon quantum dots and graphite nanocrystals in coals. Nanoscale.

[33] Chen BB (2016). A large-scale synthesis of photoluminescent carbon quantum dots: a self-exothermic reaction driving the formation of the nanocrystalline core at room temperature. Green Chem..

[34] Wang D-Y (2015). Photoluminescence quenching of graphene oxide by metal ions in aqueous media. Carbon.

[35] Guo Y, Wang Z, Shao H, Jiang X (2013). Hydrothermal synthesis of highly fluorescent carbon nanoparticles from sodium citrate and their use for the detection of mercury ions. Carbon.

[36] Zhang J, Yang L, Yuan Y, Jiang J, Yu S-H (2016). One-Pot Gram-Scale Synthesis of Nitrogen and Sulfur Embedded Organic Dots with Distinctive Fluorescence Behaviors in Free and Aggregated States. Chemistry of Materials.

[37] Qu K, Wang J, Ren J, Qu X (2013). Carbon Dots Prepared by Hydrothermal Treatment of Dopamine as an Effective Fluorescent Sensing Platform for the Label-Free Detection of Iron(III) Ions and Dopamine. Chemistry – A European Journal.

[38] Wu ZL (2013). One-pot hydrothermal synthesis of highly luminescent nitrogen-doped amphoteric carbon dots for bioimaging from Bombyx mori silk - natural proteins. Journal of Materials Chemistry B.

[39] Liu Y (2015). One-step synthesis of robust nitrogen-doped carbon dots: acid-evoked fluorescence enhancement and their application in Fe3 + detection. Journal of Materials Chemistry A.

[40] Yang G, Wan X, Su Y, Zeng X, Tang J (2016). Acidophilic S-doped carbon quantum dots derived from cellulose fibers and their fluorescence sensing performance for metal ions in an extremely strong acid environment. Journal of Materials Chemistry A.

[41] Yu J, Xu C, Tian Z, Lin Y, Shi Z (2016). Facilely synthesized N-doped carbon quantum dots with high fluorescent yield for sensing Fe3+. New Journal of Chemistry.

[42] Xu J, Zhou Y, Liu S, Dong M, Huang C (2014). Low-cost synthesis of carbon nanodots from natural products used as a fluorescent probe for the detection of ferrum(iii) ions in lake water. Analytical Methods.

[43] Li G, Lv N, Bi W, Zhang J, Ni J (2016). Nitrogen-doped carbon dots as a fluorescence probe suitable for sensing Fe3 + under acidic conditions. New Journal of Chemistry.

[44] Zhang Y-L (2013). Graphitic carbon quantum dots as a fluorescent sensing platform for highly efficient detection of Fe3 + ions. RSC Advances.

[45] Miao P, Tang Y, Han K, Wang B (2015). Facile synthesis of carbon nanodots from ethanol and their application in ferric(iii) ion assay. Journal of Materials Chemistry A.

[46] Liu M, Xu Y, Niu F, Gooding JJ, Liu J (2016). Carbon quantum dots directly generated from electrochemical oxidation of graphite electrodes in alkaline alcohols and the applications for specific ferric ion detection and cell imaging. Analyst.

[47] Wang D, Wang L, Dong X, Shi Z, Jin J (2012). Chemically tailoring graphene oxides into fluorescent nanosheets for Fe3 + ion detection. Carbon.

[48] Gong X (2015). Facile synthesis of nitrogen-doped carbon dots for Fe3 + sensing and cellular imaging. Analytica Chimica Acta.

[49] Zheng M (2016). One-Pot To Synthesize Multifunctional Carbon Dots for Near Infrared Fluorescence Imaging and Photothermal Cancer Therapy. ACS Applied Materials & Interfaces.

[50] Ge J (2015). Red-Emissive Carbon Dots for Fluorescent, Photoacoustic, and Thermal Theranostics in Living Mice. Advanced Materials.

[51] Zeng Y-W (2015). N, S co-doped carbon dots with orange luminescence synthesized through polymerization and carbonization reaction of amino acids. Applied Surface Science.

