# Supplementary material for: High Quantum Yield Green-Emitting Carbon Dots for Fe(ІІІ) Detection, Biocompatible Fluorescent Ink and Cellular Imaging
Source: Sci Rep. 2017 Nov 1;7:14866. doi: 10.1038/s41598-017-15054-9 (PMC5665951; doi:10.1038/s41598-017-15054-9)
Supplement: Supplementary file 1 — Supporting information [file 41598_2017_15054_MOESM1_ESM.doc]

**High Quantum Yield Green-Emitting Carbon Dots for Fe(ІІІ) Detection, Biocompatible Fluorescent Ink and Cellular Imaging**

**Waheed Ullah Khan,^1^ Deyin Wang,^1^* Wei Zhang,^2^ Zuobin Tang,^1^ Xinlong Ma,^1^ Xin Ding, ^1^ Shanshan Du, ^1^ and Yuhua Wang,^1^***

^1^School of Physical Science and Technology, Lanzhou University, Lanzhou 730000, P.R. China.

^2^School of Basic Medical Sciences, Lanzhou University, Lanzhou 730000, P.R. China

**Fluorescence sensors for Fe^3+^ detection**

The detection of Fe^3+^ was performed at room temperature in aqueous solution of green-emitting CDs at pH 7.2. The standard stock solution of Fe^3+^ was prepared by dissolving the appropriate amount of FeCl_3._6H_2_O. To evaluate the sensitivity towards Fe^3+^different concentration of (25 µM-300 µM) Fe^3+^ were added into the aqueous solution of green-emitting CDs and the mixed solution were sonicated for one minute before photoluminescence measurements. The photoluminescence spectra were recorded with an excitation wavelength of 420 nm.

**Quantum Yield measurement**

Quantum yield (QY) measurement was performed according to the slope method.^1,2^ Rohdmaine 6G dispersed in ethanol (QY 95%) was employed as standards. The absorbance of the solution for green-emitting CDs and Rhodamine 6G were kept below 0.06. The QY of the prepared green-emitting CDs was calculated according to the following equation (1).

φ_x_= φ_st_ (K_x_/K_st_) (η^2^ _x_/η^2^ _st_)…………………(1)

Where φ is the quantum yield, K is the slope of the fitted line and η is the refractive index of the solvent. The subscript “x” refers to the testing sample and “st” refers to the standards (Rhodamine 6G). The value of refractive index is 1.33 and 1.36 for water and ethanol respectively.

**Cytotoxcity assay**

The cytotoxicity of the green-emitting CDs against HeLa cells were evaluated by the standard MTT (3-(4,5-dimethylthiazol-2yl)-2,5-diphenyltetrazolium bromide) assay. Typically, HeLa cells were seeded in culture dishes (96-well cell plats) in Dulbec co's Modified Eagle's Medium (DMEM) with a density of 4 x 10^4^ cells mL^-1^ for 24 hours. After incubation for 24 h at 37 °C with 5% CO_2_, the culture medium was replaced and then cells were treated with (DMEM) containing various concentrations of green-emitting CDs (100-500 μg mL^-1^) for another 24 h. At the end of the incubation process, the culture medium was removed, and 20 μL of MTT (5.0 mg mL^-1^ in PBS) was added to each dish. After additional 4 hours incubation, the growth medium was removed and 150 μL of DMSO was added into each well to dissolve MTT. Finally, the optical absorbance of each sample was recorded using a microplate reader (Imark 168-1130, Biorad, USA) at a wavelength of 450 nm.

**Cellular imaging**

Cellular imaging of the as obtained green-emitting CDs was tested by the use of HeLa cells. 2.0 mL of HeLa cells in DMEM medium at an initial density of 4 x 10^4^ cell mL^-1^ were seeded in each wall and cultured at 37 °C for 24 h under 5% CO_2_. The dispersion of the green-emitting CDs was prepared in DMEM medium with a concentration of 200 μg mL^-1^. Cells were cultured with the dispersion of green-emitting CDs for 2 h and then washed three times with phosphate buﬀer solution (PBS) to remove the extra green-emitting CDs. At last the sample was observed with Scanning Confocal Microscope (TCS SP5II, Leica, Germany).


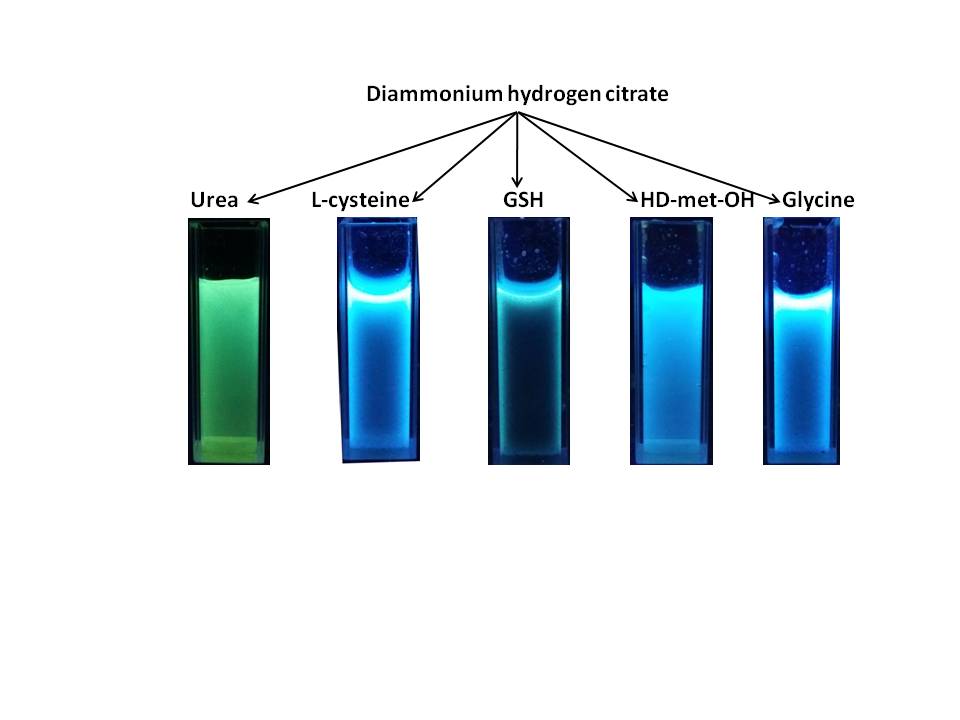


**Figure S1**. Photographs of carbon dots synthesized by using diammounium hydrogen citrate with other materials under 365 nm lamp irradiation. From the left to the right is urea, L-cystiene, L-glutathione (GSH), HD-met-OH and glycine respectively.

**
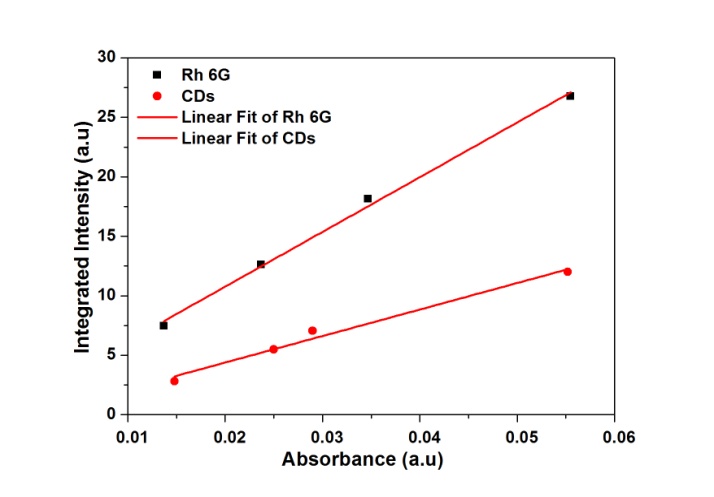
**

|  | Green-emitting CDs | | | | Rhodamine 6G | | | |
| --- | --- | --- | --- | --- | --- | --- | --- | --- |
| Abs | 0.0148 | 0.025 | 0.029 | 0.0522 | 0.0137 | 0.0237 | 0.0347 | 0.0555 |
| Integrated Intensity (10^6^) | 2.81 | 5.48 | 7.06 | 12.06 | 7.469 | 12.6 | 18.14 | 26.76 |
| Excitation (λ_ex_) | 420 nm | | | | 420 nm | | | |
| Slope | 2.2x10^8^ | | | | 4.5 x10^8^ | | | |
| QY (%) | 46.4 | | | | 95 | | | |

**Figure S2**. Plots of integrated intensity of green-emitting CDs and rhodamine 6G as a function of optical absorbance at 420 nm and relevant data in ethanol.


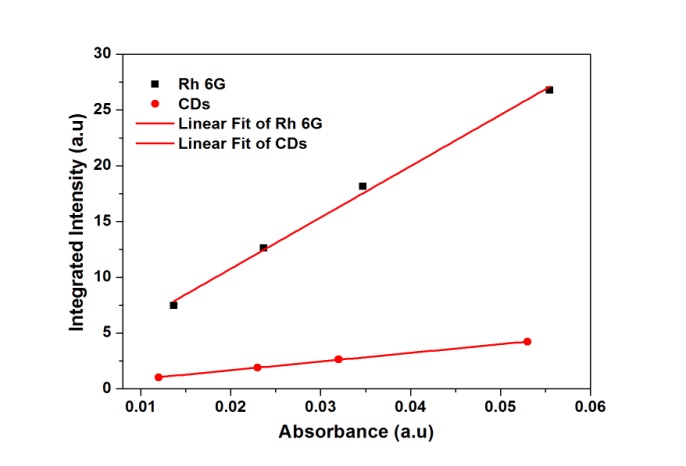


|  | Green-emitting CDs | | | | Rhodamine 6G | | | |
| --- | --- | --- | --- | --- | --- | --- | --- | --- |
| Abs | 0.012 | 0.023 | 0.032 | 0.053 | 0.0137 | 0.0237 | 0.0347 | 0.0555 |
| Integrated Intensity (10^6^) | 1.03 | 1.904 | 2.64 | 4.219 | 7.469 | 12.6 | 18.14 | 26.76 |
| Excitation (λ_ex_) | 420 nm | | | | 420 nm | | | |
| Slope | 7.7x10^7^ | | | | 4.5 x10^8^ | | | |
| QY (%) | 15.5 | | | | 95 | | | |

**Figure S3**. Plots of integrated intensity of green-emitting CDs and rhodamine 6G as a function of optical absorbance at 420 nm and relevant data in DI water.


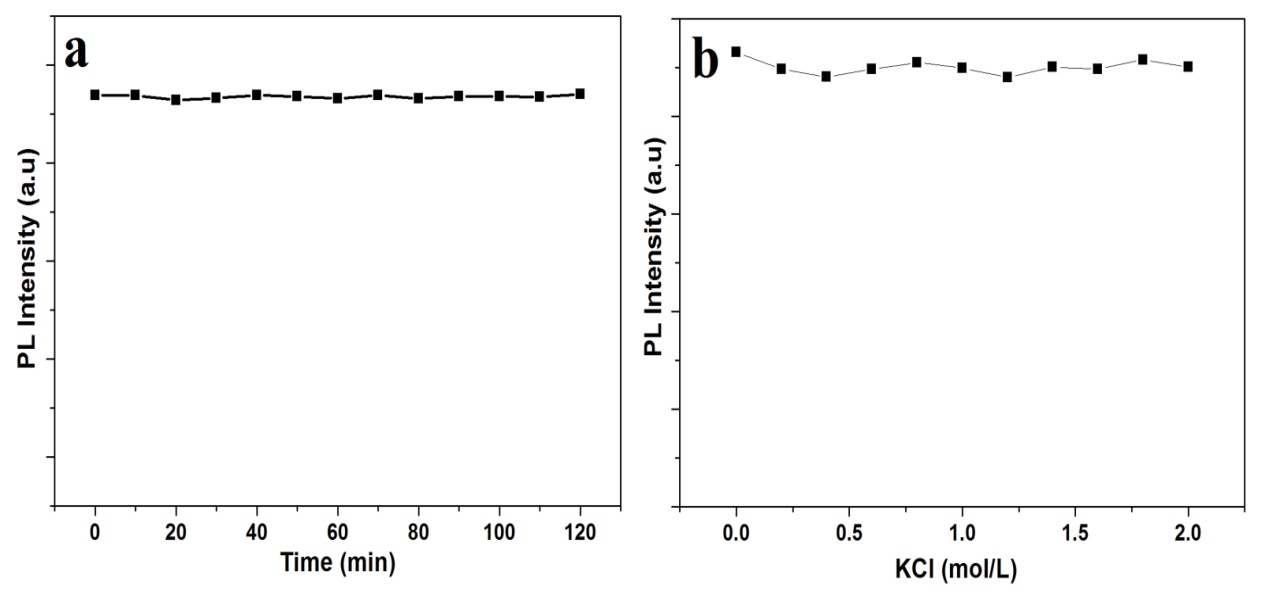


**Figure S4.** (a) Photo stability of the green emitting CDs irradiated by a 365 nm UV lamp at various time and (b) the effect of salt (KCl) concentration on the PL intensity of the the green emitting CDs.


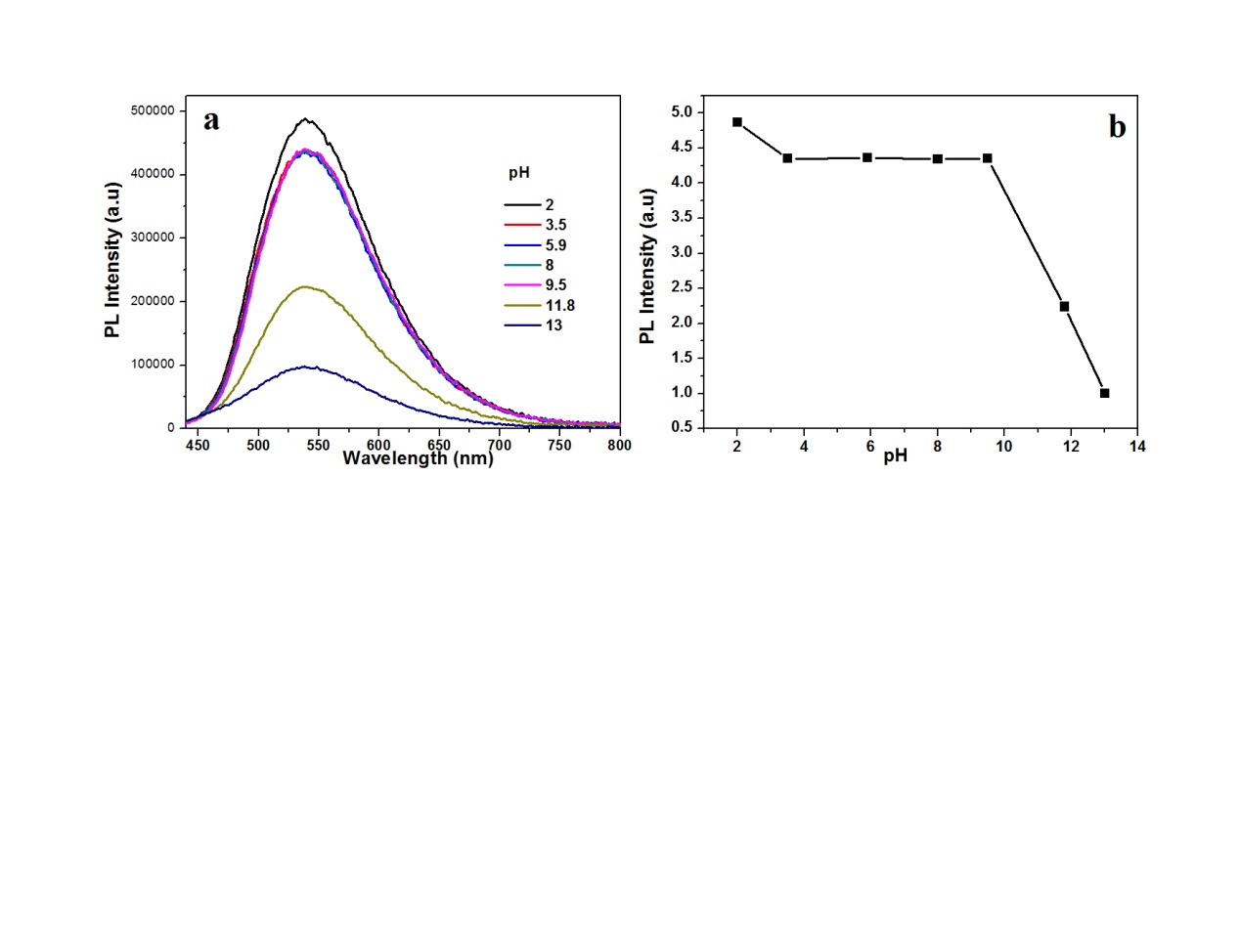


**Figure S5.** (a) PL spectra of the green-emitting CDs under different pH values. (b) PL intensity of the green emitting CDs at 537 nm at different pH values.


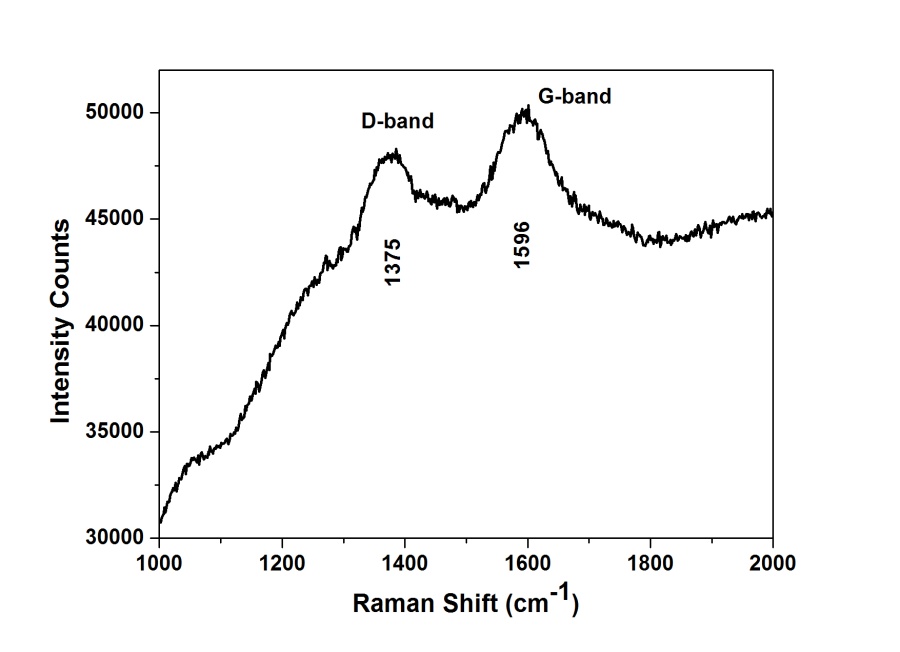


**Figure S6.** Raman spectrum of the obtained green-emitting CDs.
